# A ^1^HNMR-Based Metabonomics Study of Postmenopausal Osteoporosis and Intervention Effects of Er-Xian Decoction in Ovariectomized Rats

**DOI:** 10.3390/ijms12117635

**Published:** 2011-11-08

**Authors:** Liming Xue, Yin Wang, Lei Liu, Lu Zhao, Ting Han, Qiaoyan Zhang, Luping Qin

**Affiliations:** 1 School of Pharmacy, Second Military Medical University, No. 325 Guohe Road, Shanghai 200433, China; E-Mails: lm.xue@hotmail.com (L.X.); 337589971@qq.com (L.L.); zhaolu850813@163.com (L.Z.); hantinglh1@hotmail.com (T.H.); 2 Department of Pharmaceutics, No. 455 Hospital of CPLA, Shanghai 200052, China; E-Mail: wangsbox@hotmail.com; 3 Department of Pharmacy, Fujian University of Traditional Chinese Medicine, Fuzhou 350108, China

**Keywords:** ^1^HNMR, metabonomics, ovariectomized rats, postmenopausal osteoporosis, Er-Xian Decoction

## Abstract

A metabonomics method using ^1^H nuclear magnetic resonance spectroscopy (^1^HNMR) was applied to obtain a systematic view of the development and progression of postmenopausal osteoporosis. Using partial least squares discriminant analysis (PLS-DA), 26 and 34 characteristic resonances were found respectively in urine and plasma of ovariectomized rats (Variable importance, VIP value ≥1.0), and the significant altered metabolites identified in the plasma and urine were 10 and 9, respectively. Changes in these metabolites were related to the pathways of lipid, energy and amino acid metabolism, some of which involved the oxidative system. The described method was also used to analyze the therapeutic effects of Er-Xian Decoction (EXD), a traditional Chinese medicine widely used in the clinical treatment of osteoporosis in China. The results showed that EXD administration could provide satisfactory effects on osteoporosis through partially regulating the perturbed pathways of lipid, energy and amino acid metabolism and improving the anti-oxidative ability.

## 1 Introduction

Postmenopausal osteoporosis, which is believed to be associated with ovarian hormone deficiency, is by far the most common cause of age-related bone loss [1]. With the reduction in estrogen levels, there is an increase in bone breakdown related to bone formation, microarchitectural deterioration and decreased bone mass [2]. Approximately 40 percent of women over 50 years suffer from osteoporotic fracture during their lifetime [3]. Estrogen replacement therapy (ERT) has been considered the most effective for the prevention and treatment of postmenopausal osteoporosis. However, an investigation [4,5] showed that estrogen could lead to higher occurrences of breast carcinoma, endometrial cancer and cardiovascular diseases. To develop a new substance with fewer undesirable side effects based on the pathological mechanism of postmenopausal osteoporosis, the biological processes of the condition need to be further investigated to find new biomarkers and illuminate the disease mechanism.

Medicinal treatment of postmenopausal osteoporosis in China not only includes modern medicines but traditional Chinese herbal drugs. Er-Xian Decoction (EXD), a popular Chinese medicinal formula comprising *Epimedium brevicornum* Maxim. (Berberidaceae, whole herb), *Curculigo orchioides* Gaertn. (Amaryllidaceae, rhizome), *Anemarrhena asphodeloides* Bge. (Liliaceae, rhizome) *Phellodendron chinense* Schneid. (Rutaceae, bark), *Morinda officinalis* How. (Rubiaceae, root), *Angelica sinensis* (Oliv.) Diels (Umbelliferae, root), and in a compositional ratio of 9:9:6:6:9:9 have been used for the treatment of osteoporosis, menopausal syndrome and age-associated diseases in the past 50 years. A systemic review and Meta-analysis of 677 participants involved in 5 clinical investigations indicated that EXD was clinically effective in relieving menopausal syndrome via increasing the circulatory estradiol level [6,7]. EXD was also shown to have positive effects on bone in ovariectomized rats [8], and could increase proliferation and activity of alkaline phosphates (ALP) of primary osteoblastic cells, and decrease the formation, differentiation and tartrate-resistant acidic phosphates (TRAP) activity of osteoclast-like multinucleated cells (MNCs) induced from bone marrow cells of rat [9]. However, the action mechanism of EXD is not clearly defined due to complexity and interaction of the active components.

Metabonomics, which is defined as the quantitative measurement of the dynamic multiparametric metabolic response of living systems to pathophysiological stimuli or genetic modification, has been used to characterize the biochemical pattern of endogenous metabolic composition in biological samples, and detect the physiopathological response to a drug and disease-induced disturbance in an endogenous metabolic network [10,11]. Estrogen deficiency in postmenopausal or ovariectomized women may cause changes in many physiological processes, leading to occurrences of cardiovascular disease, obesity, osteoporosis and alteration of metabolites in blood and urine. In recent years, some studies have reported altered metabonomics in ovariectomized rats, a common model for assessing osteoporosis. The metabonomic analysis based on ^1^HNMR in ovariectomized rats found that ovareictomy for 5 months lead to the elevated level of lactate, acetone and ethanol, and decreased level of glucose, choline/phosphatidylcholine, alanine, high density lipoprotein/low density lipoprotein (HDL/LDL), very low density lipoprotein/low density lipoprotein, fatty acid in the plasma of rats [12]. A GC-TOF/MS-based metabolomic investigation indicated that ovariectomy caused elevation in cholesterol, glycerol, glucose, arachidonic acid, glutamic acid, glycine, and cystine, and decline in alanine of serum in 12-month obese rats [13]. The metabonomic analysis using UPLC-q-TOF-MS demonstrated that the level of fatty acids such as arachidonic acid (AA), eicosapentaenoic acid (EPA) and cholecalciferol was increased, and the level of ergocalciferol was decreased in serum of 12-week rats with ovariectomy-induced estrogen deficiency [14]. However, there is no investigation reporting changes in bone metabolism tightly associated with estrogen withdrawal. The three detection methods applied in metabonomic analysis (^1^HNMR, GC-MS and UPLC) have their advantages and limitations respectively. The ^1^HNMR analytical method has the advantage of the simple fast sample preparation process, short measurement time, and advanced data analysis methods. In the present study, a ^1^HNMR-based metabonomic strategy was applied to find changed metabolites in urine and blood of osteoporotic ovariectomized rats with the purpose of gaining insights into the pathological process of postmenopausal osteoporosis. In addition, this is also the first study to investigate possible reverse effects of EXD and explore its action mechanism using a metabonomic approach.

## 2. Experimental

### 2.1. Materials

The six plant materials of EXD were obtained from Hua Yu Pharmarceutical Company in Shanghai, China, and identified by Prof. H.C. Zheng of the Department of Pharmacognosy, School of Pharmacy of the Second Military Medical University in Shanghai, China. Their voucher specimens are available in the herbarium of this Department. A 1500 g mixture of six crud drugs in EXD with the weight ratio of 9:9:6:6:9:9 was extracted by decocting the mixed herbs with 10× (v/w) distilled water at 100 °C for 2 h. After filtration, the residue was boiled for an additional 1 h. Filtrates were mixed together, lyophilized with a freeze drier (Labconco, Preezone), and kept at 4 °C. The yield of the dried extract from the starting crude materials was 10%. The EXD extracts resolved to 60 mg/mL in water were administered orally to rats at a volume of 1 mL/100 g body weight which equal to human daily dosage.

The assay kits for alkaline phosphatase (ALP, A059-1), tartrate-resistant acid phosphatase (TRAP, A058), superoxide dismutase (SOD, A001), glutathione peroxidase (GPX, A005), and malondialdehyde (MDA, A003) were purchased from Nanjing Jiancheng Bioengineering Institute (Nanjing, China). RIA kits for measurement of estradiol levels were purchased from China Institute of Atomic Energy (Beijing, China, S20103078).

### 2.2. Animals

Twenty four female Sprague-Dawley (SD) rats aged 12 weeks were purchased from Slacom Experimental Animal Company (Shanghai, China,) and acclimated to conditions for 1 week before the experiment. The experimental animals were housed in an air-conditioned room with 12 h/12 h light-dark illumination cycles at constant temperature 25 ± 2 °C and humidity (50% ± 10%). Food and drinking water were supplied *ad labitum*. The rats were weighed weekly during the experimental period.

### 2.3. Establishment of the Osteoporotic Model and Drug Administration

The osteoporotic model was established by removing the bilateral ovaries of rat for 12 weeks. Of the 24 female SD rats, 8 were sham-operated and treated with deionized water as the aging control group (sham). The remaining rats were bilaterally ovariectomized and equally randomized into two groups. They were treated with vehicle (deionized water) or EXD extract (600 mg·kg^−1^·d^−1^, ig) for 12 weeks, starting from day one of surgery. Success of ovariectomy was confirmed at necropsy by failure to detect the ovarian tissue and by observation of marked atrophy of the uterine horns. At the end of the treatment, the femurs were cleaned off from adhering soft tissues, and then enclosed with PBS-saturated gauze and stored in a freezer at −80 °C. Bone mineral density (BMD) was determined by dual-energy X-ray absorptiometry (LUNAR Co. Ltd., USA) using the small animal scan mode.

### 2.4. Sample Collection

The animals were weighed weekly during the experimental period. At the end of the treatment, the rats were housed individually in metabolic cages without providing food for a day. Urine samples were collected from 20:00 p.m. to 8:00 a.m. on the following day. The blood sample was collected via abdominal aorta puncture, stabilized with sodium heparin, and then centrifuged at 5000 rpm for 10 min. All samples were then stored at −80 °C for use. The uterus was removed and weighed immediately. ALP, TRAP, SOD and GSH activities and MDA content were measured on an automatic analyzer (Ciba-Corning 550, USA) using diagnostic reagent kit *in vitro* determination. The level of estradiol (E_2_) was determined using a specific and sensitive double-antibody RIA kit on a gamma-ray counter. This experiment was approved by the Bioethic Committee of the Second Military Medical University, and the procedures of the experiment were performed strictly according to the generally accepted international rules and regulations.

### 2.5. ^1^HNMR Spectroscopy

Urine or plasma samples were defrosted and centrifuged at 10,000 rpm for 10 min before ^1^HNMR detection. 400 μL supernatant was transferred into NMR tubes (diameter, 5 mm), with addition of 200 μL PBS buffer solution, 100 μL D_2_O and 50 μL DSS (2, 2-dimethylsilapentane-4-sulfonic acid, 1 mM final concentration, internal standard). ^1^HNMR measurement was made on a Bruker AVANCE II 600 spectrometer at 600. 13 MHz ^1^H frequency (Bruker Spectrospin AG, SWISS). ^1^HNMR spectra of the urine and plasma samples were acquired using a solvent pre-saturation pulse sequence to suppress the residual water resonance. Free induction decays (FIDs) were collected at 64 K data points, at 300 K, with a spectral width of 7200 Hz and an acquisition time of 2.04 s, giving a total pulse recycle delay of 3.04 s. The data were zerofilled by a factor of 2, and FIDs were multiplied by an exponential weighting function equivalent to a line broadening of 0.3 Hz prior to Fourier transformation.

### 2.6. Multivariate Statistical Analysis

The phase and baseline of all ^1^HNMR spectra were manually corrected using MestReNova software (Mestre, Inc.). The range of *δ* 10–0 ppm in standard ^1^HNMR spectra were automatically segmented into 500 regions at 0.02 ppm intervals with δ 4.5–5.2 ppm (spectral region incorporating water) set to zero integrals. The data of each sample were normalized to total area to correct for the NMR response shift. The final data were submitted to SIMCA-P (Version 11.0, Umetrics, Umea, Sweden) software package for multivariate analysis. The data were mean-centered and pareto-scaled before this analysis. Then Partial least square discriminant analysis (PLS-DA), which has been widely used in metabonomic studies [15–17], was performed to clarify which chemical shift regions carry the separating information. Cross-validation was used to validate the PLS-DA model according to the default settings in the software.

### 2.7. Statistics Analysis

All numerical data are presented as mean ± SD from replicate experiments. Student’s *t*-test and one-way analysis of variance (ANOVA) were used to evaluate differences between groups. The significance level was set at *P* < 0.05 for all tests. Statistical analysis was performed using PASW version 18.0 software (SPSS Inc., Chicago, IL, USA).

## 3 Results and Discussion

### 3.1. Histopathology

Ovariectomy induces bone loss, body weight gain and uterine weight reduction. As shown in Table 1, there was no significant difference in the initial body weight between the three groups at the beginning of the study. At the end of the study, there was a significant increase in body weight in OVX and OVX + EXD animals. As expected, the mean uterine weight of OVX animals was significantly lower than that of sham controls. It is noteworthy that the mean uterine weight of OVX + EXD animals was identical to that of OVX animals. In addition, ovariectomy induced a decrease in serum E_2_, and EXD treatment improved E_2_ level compared with the OVX model group. Serum TRAP is a biochemical marker of bone resorption, and ALP is a marker of bone formation. Our results showed that ovariectomy induced high bone turnover in rats, the femur BMD significantly decreased after 12 weeks of ovariectomy compared with sham rats, while serum ALP and TRAP levels significantly increased. Administration of the EXD extract significantly increased BMD of the femur, and decreased serum ALP and TRAP levels. These results indicate that ovariectomy induces osteoporosis of rats, and the EXD extract improved BMD of the femur and decreased bone loss induced by ovariectomy.

### 3.2. Metabonomic Analysis of Osteoporosis Based on ^1^HNMR Metabolic Profiles in Rat Urine and Plasma

**Figure 1:**
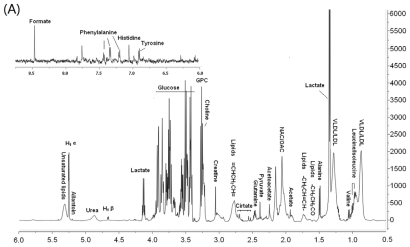

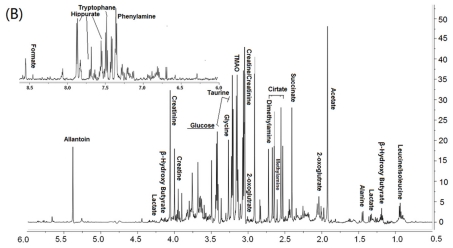
Representative 600 MHz ^1^H nuclear magnetic resonance spectroscopy (^1^HNMR) spectra (*δ* 10–0.5 ppm) of plasma (**A**) and urine sample (**B**) in sham rats. GPC: glycerophosphatide choline; TMAO: Trimethylanine-oxide; OAC: *O*-acetyl-glucoprotein; NAC: *N*-acetyl-glucoprotein.

**Figure 2:**
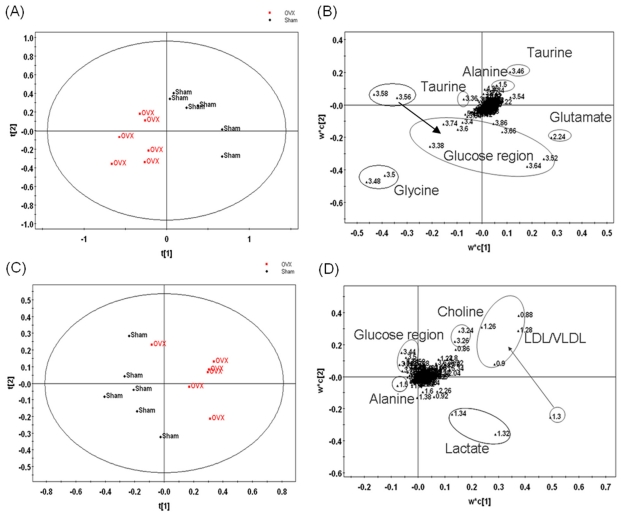
PLS-DA scores plot (**A**) and corresponding loadings plot (**C**) are based on the ^1^H NMR spectra of the plasma samples from Sham (■) and OVX(◆); Scores plot (**B**) and corresponding loadings plot (**D**) are based on the ^1^HNMR spectra of the urine samples from Sham(■) and OVX(◆).

Typical ^1^HNMR spectra of plasma and urine samples are shown in Figure 1A,B respectively, with major metabolites in the integrate regions assigned. The resonance assignments were made according to published reports and the Metabonomics Toolbox (http://www.hmdb.ca) [18]. PLS-DA was used to analyze all the ^1^HNMR metabolite profiles, and the separation was readily detected. The reliability of the established PLS-DA models was evaluated by the explained variation *R*^2^ and the predicted total variation *Q*^2^ which is calculated by cross-validation. The expected *R*^2^ and *Q*^2^, highly dependent on their application fields, should respectively be more than 0.5 and 0.4 for significant biological model[19]. In our established model for postmenopausal osteoporosis of rats, the value of R^2^ and *Q*^2^ were above 0.6, indicating that the PLS-DA model was established successfully (Table 2). The scores plots of urine samples (Figure 2A) and plasma samples (Figure 2C) showed well separation between sham and OVX groups, indicating that the metabolic characteristics of the two groups were markedly different. The corresponding loading plot of urine (Figure 2B) and plasma (Figure 2D) shows the contribution of variables to the differences between the OVX and sham groups. The farther the variable deviates from the origin, the higher value of variable importance projection (VIP) will be obtained. When VIP value is ≥1.0, the variable can be considered as a contributor for the classification of OVX and sham groups. Twenty six characteristic resonances in urine and 34 in plasma were found in the ovariectomized rats (VIP value ≥1), and 10 significant altered metabolites in plasma and 9 in urine were identified as listed in Table 3.

### 3.3. Biological Significance of the Potential Biomarkers in the Osteoporotic Rats

Ten endogenous metabolites in plasma and nine endogenous metabolites in urine were selected as potential biomarkers from VIP and loading plots together to get a complete picture. To get more information on the reference of these metabolites, we searched the KEGG PATHWAY Database (http://www.genome.jp/kegg/) [31], and found that the altered metabolites are mainly involved in lipid, energy and amino acid metabolism.

As shown in Table 3, the increased plasma levels of low density lipoprotein (LDL), choline, lipids and glycerophosphatide choline in ovariectomized rats indicated that the biological mechanism of osteoporosis is associated with lipid metabolism. The findings that there is a connection between lipid metabolism and bone remodeling proved by other investigations. OVX-induced estrogen withdrawal resulted in both bone loss and an increase in fat [32]. Osteoporotic postmenopausal women have a higher level of lipids in blood [20], and statin, a lipid-lowering drug, increased BMD in clinical studies [21,33]. Excess fat induces bone loss, and free fatty acid is a strong candidate for the cause of bone loss by increasing osteoclast formation [23]. Therefore, the changes of lipids in blood may predict the abnormality of bone metabolism.

We can see from Table 3 that the levels of lactate and glucose, which are associated with energy metabolism, were significantly increased in plasma and urine of the ovariectomized rats, indicating that ovariectomy affects the energy metabolism and utilization. It has been reported that ovariectomy and etrogen deficiency led to decreased energy expenditure[27], increased glucose level and increased visceral adipose tissue [34,35]. Long-term estrogen deficiency may lead to reduction of insulin secretion, thus weakening the ability of controlling glucose level in ovariectomized rats [26]. The elevated glucose concentration stimulated cellular proliferation during the development of murine osteoblasts [25], while inhibiting calcium uptake. Extracellular ATP and other nucleotides can exert impressive stimulatory effects on the formation and activity of osteoclasts (bone-resorbing cells), in addition to inhibiting bone formation by osteoblasts [24]. It may be speculated that ovariectomy and estrogen deficiency led to disturbance of energy metabolism, and further the imbalance of bone remodeling.

In the formation of postmenopausal osteoporosis, the amino acid metabolism is also definitely disturbed. This can be seen from the urine metabolite profile of OVX rats. As shown in Table 3, the levels of glycine, allantoin and hippurate were upregulated, and taurine, glutamate and alanine were down-regulated in the urine of OVX rats, some of which were involved in the bone remodeling. Taurine, which is found in a high concentration in bone cells, is thought to inhibit the formation and survival of osteoclasts [29,36], induce cell proliferation and differentiation in human osteoblast (HOB) cells [30]. In the present study, urine taurine level in the OVX rats decreased dramatically. This might indicate the increased utilization or decreased excretion of taurine because of the stress reaction of ovariectomy. l-glutamate (Glu), which is an excitatory amino acid neurotransmitter in the mammalian central nervous system, could markedly inhibit osteoclastogensis, prevented BMD from decreasing in ovariectomized mice [28]. In our study, urine glutamate was reduced in the ovariectomized rats, indicating that ovariectomy resulted in decreased glutamate production, which further affected the bone remodeling. Urine taurine and glutamate may prove to be useful biomarkers for evaluating the risk of osteoporosis and fracture.

### 3.4. Metabonomic Analysis of EXD Treatment

**Figure 3:**
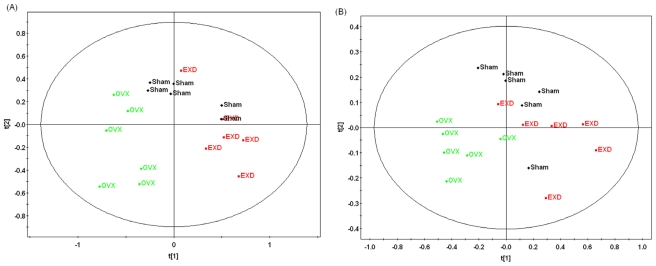
Scores plot from PLS-DA of ^1^HNMR data of urine (**A**) and plasma (**B**) samples obtained from control rats, OVX rats and EXD treatment rats.

**Figure 4:**
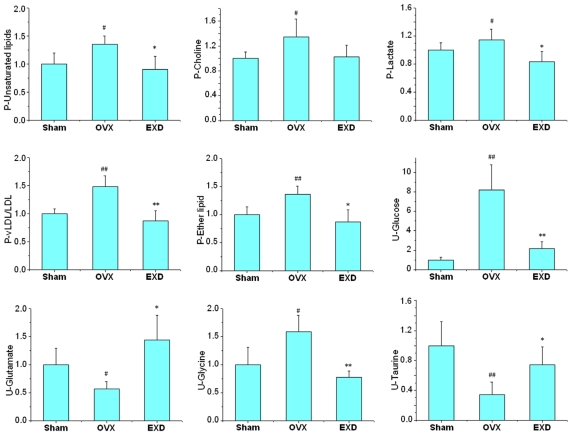
The relative levels of nine significantly altered metabolites in the rats of sham, OVX and EXD treatment groups. The percentage has been calculated with the formula: Contents in groups /content in control group × 100%. P—plasma, U—urine. ^##^ *P* < 0.01, ^#^ *P* < 0.05 compared with sham group; ** *P* < 0.01, * *P* < 0.05 compared with OVX group.

According to the histopathology results (Table 1), EXD treatment had a positive effect on the OVX model in characteristic osteoporosis-related indexes, including BMD, ALP and TRAP activity. All of the values of *R*^2^ and *Q*^2^ in our established PLS-DA models are more than 0.5, so this model can be used to differentiate the OVX rats from the sham and the EXD-administrated rats by analyzing the ^1^HNMR data. As shown in Figure 3A,B, the dots of EXD treatment group were closer to sham group and far away from OVX group, which might suggest that EXD reversed the pathological process of OVX-induced osteoporosis. Most of the biomarkers in Table 3 could be reversed to normal levels or close to normal levels by EXD intervention. Nine metabolites, including lipids, choline, LDL, lactate, glucose, glutamate, glycine and taurine, which are all related to the lipid or amino acid metabolism as mentioned above, were completely reversed to normal levels by EXD (Figure 4). In addition, EXD significantly up-regulated the metabolite levels related to energy metabolism, including glycerophosphatide choline, citrates, creatine and 2-oxo-glutamate, and also increased the urine level of tryptophane, which is a precursor for serotonin. Serotonin has been shown to increase bone mass by increasing the recruitment and proliferation of osteoblasts. The results of histopathology and metabonomics demonstrated that EXD had extensive effects in the treatment of postmenopausal osteoporosis through regulating the disturbed pathways of lipid, energy and amino acid metabolisms.

### 3.5. Determination of Superoxide Dismutase Activity, Glutathione Peroxidase Activity and Malondialdehyde Content

**Figure 5:**
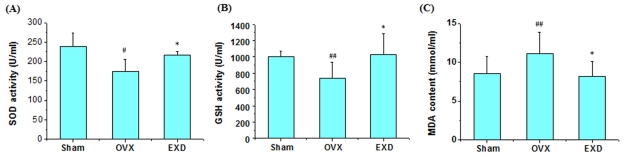
The activity of SOD (**A**), GSH (**B**) and the content of MDA (**C**) in serum of rats of sham, OVX and EXD treatment groups. ^##^ *P* <0.01, ^#^ *P* < 0.05 compared with sham group; ** *P* < 0.01, * *P* < 0.05 compared with OVX group.

The altered metabolites in plasma and urine of ovariectomized rats such as lipids, LDL, taurine and glutamate are involved in the oxidative defense system. Some studies [37,38] showed that ovariectomy induced oxidative stress, impaired the bone antioxidant system in adult rats, increased the production of lipid peroxidation and H_2_O_2_, and reduced the enzymatic antioxidants like SOD and GSH-Px in rats. This correlation of oxidative stress and bone metabolism can also be further illustrated by the correlation between plasma oxidative state and BMD. Plasma total oxidative status value is significantly higher, and plasma total antioxidant level was lower in osteoporotic patients than that in healthy controls, and there is a significant negative correlation between total oxidative value and BMD in lumbar and femoral neck region [21]. EXD had an anti-aging effect in that it raise the activities of antioxidant enzymes, such as SOD, CAT, and reduce the production of free radicals [39]. To confirm this mechanism, we evaluated the levels of SOD, GSH activity and MDA content in serum of ovariectomized rats. As shown in Figure 5A–C, serum levels of SOD and GSH in OVX model rats were significantly decreased, and the content of MDA significantly increased compared with the sham rats (*P* < 0.05), while this alteration could be reversed by the intervention of EXD treatment (*P* < 0.05), indicating that oxidation is involved in the pathological process of postmenopausal osteoporosis, and EXD prevents osteoporosis by involving the oxidative system.

## 4. Conclusion

In this study, an NMR-based metabonomic approach has been successfully established to study metabolic changes in osteoporotic ovariectomized rats and action mechanism of EXD. As a result, lipid metabolism, energy metabolism, and amino acid metabolism changed in ovariectomized rats. Some altered metabolites were involved in the oxidative defense system, and the levels of SOD and GSH were significantly reduced, and the content of MDA was increased in the serum of ovariectomized osteoporotic rats. EXD could reverse the pathological process of osteoporosis through regulating the disturbed pathway of lipid, energy and amino acid metabolism, improve the activity of SOD and GSP, and decrease the content of MAD in the serum of OVX rats. Our findings reveal distinct metabolic changes in osteoporosis, and may be helpful for further understanding of this disease. Furthermore, the detected pathway alteration may be useful for the development of new drug targets for the treatment of osteoporosis.

## Figures and Tables

**Table 1:** Osteoporosis-related indexes in control, OVX and Er-Xian Decoction (EXD) treatment group.

Group	Body weight(g)		Uterine index	E_2_ (pg/mL)	BMD (g/cm^2^)	TRAP (IU/L)	ALP (IU/L)

	Intial	Final					
Sham	251 ± 19	287 ± 10	4.0 ± 1.6	35.7 ± 2.6	0.26 ± 0.01	7.2 ± 1.9	80.6 ± 20.1
OVX	242 ± 6	346 ± 25 ##	1.1 ± 0.7 ##	16.7 ± 1.8 ##	0.25 ± 0.01 #	15.0 ± 2.6 ##	109 ± 20.2 #
EXD	253 ± 10	339 ± 32	1.8 ± 1.1	28.4 ± 2.2 **	0.26 ± 0.01 *	3.3 ± 1.4 **	86.8 ± 18.2 *

The uterine index is uterine weight /body weight × 1000;

# *P* < 0.05,

## *P* < 0.01 compared with sham group;

* *P* < 0.05,

** *P* < 0.01 compared with OVX group.

**Table 2:** Summary of the parameters for assessing quality of the partial least squares discriminant analysis (PLS-DA) model.

Model	No.α	*R*^2^*X*	*R*^2^*Y*	*Q*^2^
**OVX-Sham**				
Plasma	3	0.768	0.78	0.612
Urine	3	0.756	0.88	0.626
**OVX-Sham-EXD**				
Plasma	3	0.792	0.602	0.625
Urine	3	0.504	0.876	0.685

α No.: represent the number of principal component obtained by cross-validation. *R*^2^*X*: Variation explanation in *X; R*^2^*Y*: the variation explanation in the response to *Y; Q*^2^: Predict variation capability of compound.

**Table 3:** The identified biomarkers in ovariectomized osteoporotic rats and the reverse effects of EXD.

	Compounds	Group	δH (ppm)	a OVX	b EXD	Pathway	Physiological action in bone metabolism
Plasma	LDL/VLDL	−(CH_2_)*n*	1.28–1.32	↑	↓	PPAR signaling pathway	Lipid oxidation products (LPO) inhibit osteoblast differentiation [20,21];
		−CH_3_	0.86–0.9				Elevated LDL are associated with low BMD [22]
	Choline	N(CH_3_)_3_	3.24	↑	↓	Ether lipid metabolism	Elevated lipid inhibit bone remodeling [22,23]
	Lactate	βCH_2_	1.34	↑	↓	Glycolysis/Gluconeogenesis	ATP improve osteoclast (OC) formation and inhibit osteoblast (OB) proliferation [24]
		CH_2_O	4.14				
	Lipids	CH_2_CH=	5.3–5.34	↑	↓	Peroxisome	Lipid peroxides altered bone oxidative system [21]
	Lipids	CH_2_C=CH	2.78–2.82	↑	↓	Glycerolipids metabolism	OVX increase hepatic lipid production [19]
	Alanine	αCH_3_	1.5	↓	↑	Alanine, aspartate and glutamate metabolism	
	Acetoacetate	O=CCH_3_	2.26	↑	↓	Valine, leucine and Isoleucine biosynthesis	
	Glucose	H_4_	3.44	↑	↓	Carbohydrate metabolism	Glucose level related with OB proliferation [25], Ca uptake [26], bone formation [25,26] and lipid metabolism [23,27]
		H_2_	3.5				
		H_6_	3.86, 3.92				
	α-glucose	αCH	5.26		↑		
	Isoleucine	γ CH_3_	0.92	↓	↑	Valine, leucine and Isoleucine biosynthesis	
	Acetylglucoprotein	=OCNH	2.02–2.06	↑	↓	Glutamine and d-glutamate metabolism	Glu inhibit OC formation and increase BMD [28]
	Glycerophosphatide choline	ON(CH_3_)_3_	3.28		↑	Ether lipid metabolism	
	Creatine	NH_2_C=O	3.94		↑	Arginine and Purine metabolism	
Urine							
	Glycine	N−CH_2_	3.48–3.5	↑	↓	Glycine, serine and threonine metabolism	
	Glutamate	βCH_2_	2.24	↓	↑	Glutamate metabolism	[28]
	Glucose	H_6′_	3.74, 3.86	↓	↑	Carbohydrate metabolism	
		H_2_	3.58–3.66				
	Taurine	βCH_2_	3.46–3.44	↓	↑	Taurine and hypotaurine metabolism	Taurine inhibit the formation of OC [29] and induce OB proliferation [30]
		αCH_2_	3.36–3.38				
	Allantoin	CH	5.4	↑	↓	Purine metabolism	
	Alanine	αCH_3_	1.48–1.5	↓	↑	Alanine, aspartate and glutamate metabolism	
	β-Hydroxy Butyrate		4.28	↑	↓	Butanoate metabolism	
	Hippurate	C_6_H ring	7.86–7.9	↑	↓	Phenylalanine metabolism	
		C_2_H ring	7.58				
	Lactate	CH_2_O	4.14–4.1	↑	↓	Glycolysis/Gluconeogenesis	
		βCH_2_	1.34				
	Tryptophane	C_4_H, ring	7.5–7.52		↑	Glycine, serine and threonine metabolism	
	Citrate	CH_2_COO	2.72		↑	Citrate cycle	
	Creatine	NH_2_C=O	4.0–4.04		↑	Arginine and Purine metabolism	
	2-oxo-glutamate	OOCCH_2_	2.98, 2.08		↑	Citrate cycle	

a ↑and ↓ represent up- and down-regulation of the compound in OVX group compared with the sham group.

b ↑and ↓ represent up- and down-regulation of the compound in EXD group compared with the OVX group.
